# Basic information about memantine and its treatment of Alzheimer's disease and other clinical applications

**DOI:** 10.1002/ibra.12098

**Published:** 2023-06-06

**Authors:** Bin‐Can Tang, Ya‐Ting Wang, Jie Ren

**Affiliations:** ^1^ Department of Anesthesiology Southwest Medical University Luzhou China; ^2^ Department of Neuroscience The University of Sheffield Sheffield UK

**Keywords:** Alzheimer's disease, memantine, N‐methyl‐D‐aspartate receptor

## Abstract

Memantine is a noncompetitive moderate‐affinity strong voltage‐dependent N‐methyl‐D‐aspartate receptor antagonist. It has been used to treat Alzheimer's disease (AD) since 1989. In 2018, it became the second most commonly used drug for the treatment of dementia in the world. AD is nonreversible, and memantine can only relieve the symptoms of AD but not cure it. Over the past half‐century, memantine's research and clinical application have been extensively developed. In this review, the basic composition of memantine, the mechanism and limitations of memantine in the treatment of AD, memantine combination therapy, comparison of memantine with other drugs for AD, and clinical studies of memantine in other diseases are reviewed to provide a valuable reference for further research and application of memantine for the treatment of AD.

## Graphical abstract

This article reviews the use of memantine in the treatment of Alzheimer's disease (AD). Memantine and other drugs have limited efficacy and side effects in the treatment of AD. Combined therapy is one possible way to improve the efficacy of memantine therapy, which needs to be explored further. It is challenging to produce an entirely new drug, and it is important to examine the usefulness of memantine in other diseases.

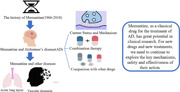

## 1 INTRODUCTION

Memantine was patented in 1966. Memantine is an antagonist of the glutamate receptor N‐methyl‐D‐aspartic (NMDA) acid receptor subtype. The neurotoxicity associated with AD and other neurodegenerative diseases can be moderated by memantine. Also, moderate to severe AD is usually treated with memantine.^1^, ^2^ Memantine alleviates the progression of AD mainly by enhancing cholinergic signaling with the inhibition of glutamate hyperactivation. However, due to the complex pathogenic mechanism of AD, it is not well studied. All drugs used to treat AD, including memantine, cannot cure AD, but can only slow down the disease process.^3^ Recently, the functions of memantine have been intensively studied. Further pharmacological effects of memantine have been revealed and applied for clinical applications, such as neuroprotection in ischemic brain injury after cardiac arrest, treatment of vascular dementia, treatment of ischemic stroke, treatment of Parkinson's disease dementia, alleviation of acute lung injury (ALI), and so on. Memantine has a wide range of effects and is of great significance for AD. Memantine is a safe and relatively economical choice for both long‐ and short‐term applications in the treatment of AD. In this review, we focus on the history, therapeutic mechanism, and limitations of memantine in the treatment of AD. We also introduce clinical research and the potential use of memantine in other diseases.

## 2 THE HISTORY OF MEMANTINE

The chemical name of memantine is 1‐Amino‐3,5‐dimethyl adamantane. Memantine belongs to amantadine in medicine classification. Memantine is clinically used as a drug, the molecular formula is C12H21N, and the average relative molecular weight is 179.3018 g/mol. The isomers and derivatives of memantine, 1‐amino‐3,7‐diethyladamantane,1‐amino‐5,7‐dimethyladamantane and 1‐amino‐3‐ethyladamantane, are also biologically active. Memantine hydrochloride is a white crystal or powder soluble in organic solvents, such as ethanol, petroleum ether, and chloroform.^4^, ^5^ Eli Lilly, the renowned American pharmaceutical company, filed a patent for a compound of memantine in 1966. However, there were no further clinical studies about memantine at that period due to its poor effect in treating hypoglycemia. Memantine was not thoroughly studied until 1982, when its central nervous system activity was discovered by Merz, a pharmaceutical company based in Germany. Memantine entered clinical trials as a treatment for dementia in 1986 and was officially launched as a product in Germany in 1989.^6^ Since then, memantine had gradually come into the public view and received a lot of attention. In November 2000, Lundbeck, as well as Merz, presented a New Drug Application (NDA) to the USA Food and Drug Administration (FDA) to treat moderate and severe AD with memantine. This application was approved on October 17, 2003.^7^ Currently, memantine has been widely accepted for AD treatment worldwide. Up to 2022, 114 memantine AD drug brands have been registered. In the United States, memantine is well known as *Namenda*.^5^

Nevertheless, memantine may induce a number of side effects. Common adverse reactions include hallucinations, confusion, headache, dizziness, high blood pressure, sleepiness, and restlessness. Less common adverse reactions include anxiety, increased muscle tone, astriction, diarrhea, nausea, anorexia, coughing, and breathing difficulties.^1^ Although memantine has some side effects, it is the only NMDA antagonist that has passed phase III clinical trials and is commercially available for treatment. Other NMDA antagonists bind very readily to NMDA receptors, causing more neuronal damage to the brain than their therapeutic effects, resulting in significant side effects and elimination. In addition, these NMDA antagonists also act on neurotransmitter receptors other than NMDA receptors, affecting signaling and causing certain side effects. Specifically, NMDA antagonists other than memantine tend to cause increased cerebral blood pressure, hallucinations, sensory loss, restlessness, and catatonia, which can cause greater harm to the organism.^8^ Memantine is the only clinically uncompetitive NMDA acid receptor antagonist that can enhance neurogenesis and regulate inflammation.^9^, ^10^ An experiment on whether memantine treatment has an effect on neuronal loss and associated behavioral deficits in a Tg4‐42 mouse model of AD showed that memantine successfully counteracted the pathological changes in the model. Memantine protects the body by reducing excitotoxicity by inhibiting the overactivation of NMDA receptors. In this process, memantine has a low affinity for NMDA receptors, so memantine does not affect the binding process of NMDA receptors to other factors and has fewer side effects than other NMDA antagonists.^11^

**Figure 1:**
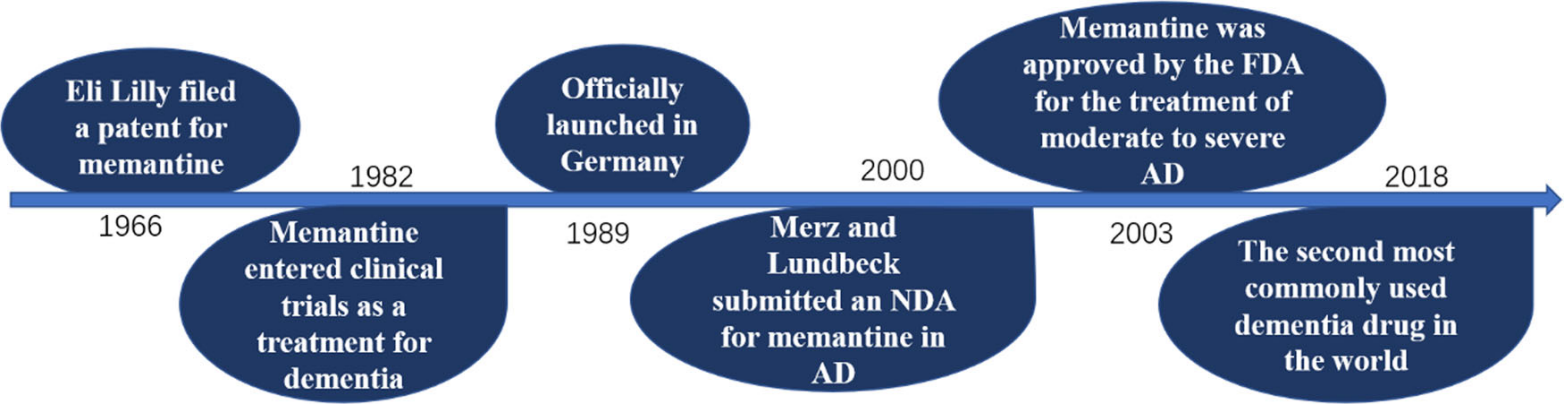
History of memantine. [Color figure can be viewed at wileyonlinelibrary.com]

To determine the trends and patterns of use of symptomatic drugs for dementia in 66 countries and regions, the researchers carried out a cross‐sectional study using large‐scale data from the Aiman International Integrated Data Analysis System database. The researchers analyzed data on dementia drug sales in 66 countries and regions between 2008 and 2018, stratifying them by income level of each country (high‐, middle‐, and low‐income countries). According to this 10‐year survey, it was found that the overall global level of awareness of dementia has increased and so has the use of symptomatic medications. It is worth noting that the developmental trend mentioned above was uneven, with the level of development in high‐income countries being much higher than in some low‐ and middle‐income countries. These low‐ and middle‐income countries are lagging behind in the level of dementia awareness as well as medical treatment. They may need international help to support the use of medications. In 2018, memantine was the second most commonly used drug for the treatment of dementia worldwide, accounting for 32.7% of total use.^12^ In conclusion, the use of allopathic dementia medications is on the rise globally. Still, some low‐ and middle‐income countries need to take measures to raise awareness of dementia to support medication use (Figure 1).

## 3 MEMANTINE THERAPY FOR AD

### 3.1 Basic information about AD

AD is the most prevalent type of dementia and one of the most common chronic diseases among the elderly, with chronic, progressive, and irreversible characteristics, affecting almost 50 million elderly individuals worldwide.^13^, ^14^ Studies suggest that 152 million people will have AD by 2050, with approximately one in every 64 people living with AD.^15^ Patients with AD have impaired cognitive function, affecting patients’ memory, mood, and mobility. The pathogenesis of AD is complex and has not been deciphered to date. Currently influential pathogenic mechanisms include extracellular deposition of amyloid plaques and intracellular neurogenic fiber tangles.^16^, ^17^ In parallel with an aging population, the morbidity and mortality rates of dementia are rapidly increasing, thus placing a huge burden on families, medical resources, the economy, and the whole of society.^18^ Currently approved drugs for the treatment of AD include cholinesterase inhibitors (donepezil, rivastigmine, and galantamine), an NMDA acid receptor antagonist (memantine), and a monoclonal antibody targeting Aβ(aducanumab), all of which are symptomatic.^19^ That is, these drugs can only provide symptomatic relief, cannot change the disease process, and cannot treat the root cause of AD. The development of drugs with the potential to alter the progression of the disease is currently a top priority.^20^

### 3.2 Current status of memantine therapy for AD

Memantine is approved for moderate and severe AD. Memantine is also a commonly used off‐label treatment of mild AD in the United States, which refers to the use of the drug for indications, doses, regimens, routes, or populations that are not within the scope of the drug's instructions approved by the drug regulatory authority.^21^ However, since 2003, only one new drug has been approved for the treatment of AD in 2021. Therefore, research on memantine for the treatment of AD is of great significance in terms of the elderly group.^16^, ^22^

According to the study, the efficacy of memantine in treating mild AD is significantly different from that in the treatment of moderate to severe AD. Memantine has no benefit for mild AD. However, memantine improves symptoms in patients with moderate to severe AD and has a good safety and tolerability profile. By alleviating symptoms such as impaired memory and cognitive decline, memantine may improve the ability to perform daily activities and reduce agitation symptoms in patients with moderate to severe AD. The clinical heterogeneity of AD makes it impossible for any single drug to have good efficacy, meaning that the best drug therapy could include the use of multiple drugs.^21^ For more than 40 years, no new drug has been approved for marketing by the FDA other than aducanumab. Aducanumab is the only drug approved by the FDA in terms of a compound that prevents brain amyloid deposition or clears existing amyloid plaques. Although aducanumab was approved, its specific therapeutic mechanism has not been fully investigated. Also, all other new drugs have failed in clinical studies.^23^ Therefore, the question of how to maximize the effect of currently approved drugs is one that we should pay attention to.

Memantine can maintain normal cellular metabolism and membrane function by inhibiting glutamate hyperactivation. It is worthwhile to continue exploring the use of memantine in AD treatment.

### 3.3 Mechanism of memantine in the treatment of AD

**Figure 2:**
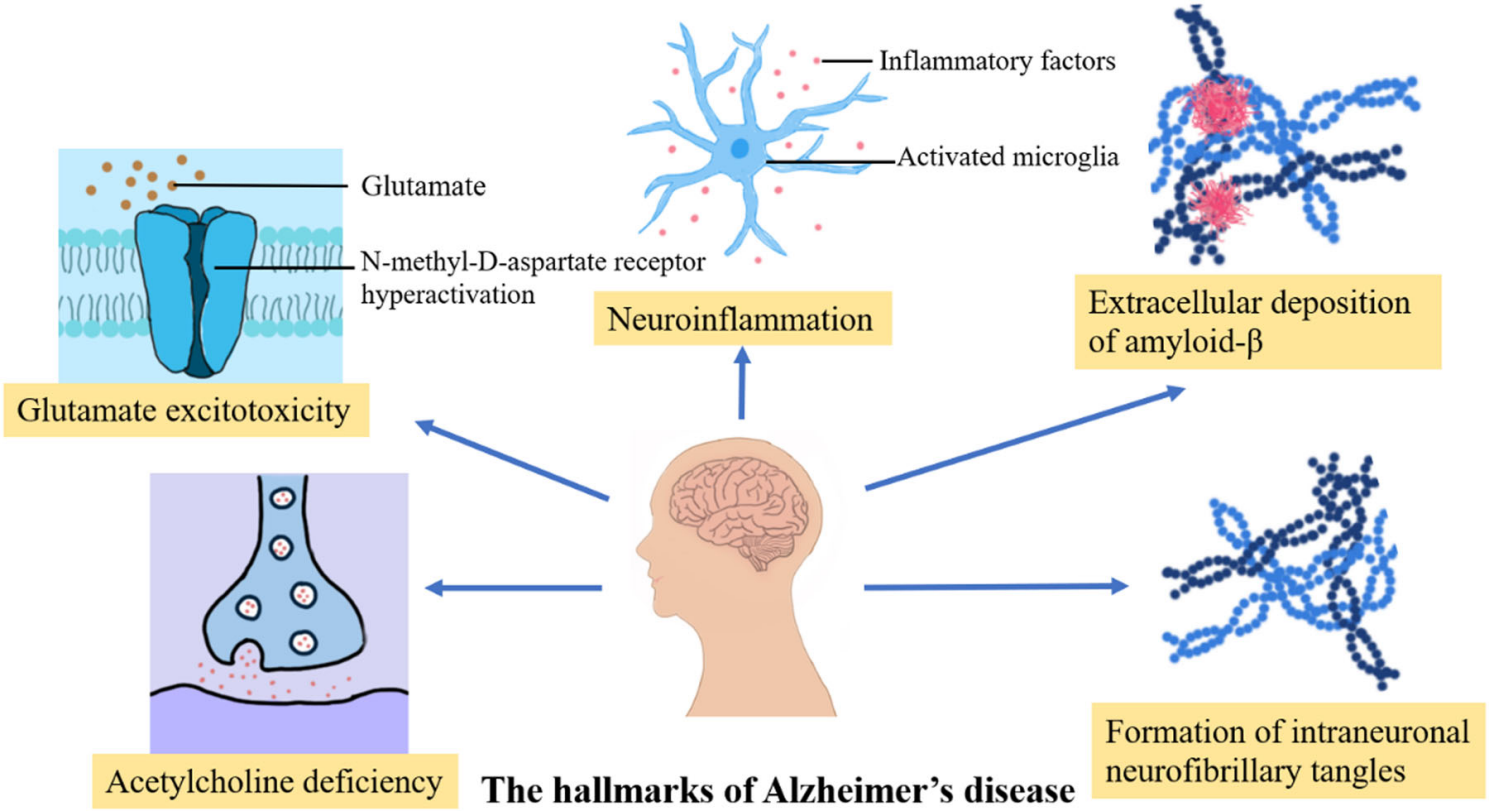
Hallmarks of Alzheimer's disease. [Color figure can be viewed at wileyonlinelibrary.com]

The treatment of AD is closely related to the neuropathological features of AD. AD has five neuropathological features, including acetylcholine deficiency, glutamate excitotoxicity, extracellular deposition of amyloid‐β (Aβ plague), formation of intraneuronal neurofibrillary tangles (NTFs), and neuroinflammation (Figure 2). Memantine therapy for AD is associated with acetylcholine deficiency and excitatory toxicity of glutamate. Patients with AD have increased acetylcholinesterase activity, increased acetylcholine breakdown, and decreased acetylcholine levels in the brain. Acetylcholine enhances memory by promoting central brain neurotransmission action, making the brain more active and excited, and positively influencing memory encoding, consolidation storage, and retrieval processes.^24^ Decreased concentrations of acetylcholine in AD patients can lead to cognitive and behavioral dysfunction. Memantine alleviates some of the dysfunction of cognition and behavior by enhancing cholinergic signaling.^25^ Glutamate is one of the major neurotransmitters in the mammalian brain. It plays an important role in post‐excitatory synaptic transmission in the nervous system and is capable of binding to a variety of postsynaptic receptors, including NMDA receptors, AMPA receptors, and others. These receptors are cationic channels that allow positively charged ions such as Na^+^, K^+^, and sometimes Ca^2+^ to enter the post‐synaptic cell, leading to depolarization and, thus, excitation of the neuron. Also, glutamate is very important for information processing and neural development. The NMDA receptor is a glutamate gate channel that mediates synaptic plasticity by acting as a coincidence detector.^26^ Hyperactivation of NMDA receptors leads to glutamate hyperactivation, which leads to excitotoxicity and may enhance local neuronal vulnerability.^27^ This is consistent with the neuropathological symptoms of AD. Memantine acts as a noncompetitive NMDA receptor antagonist with low to moderate affinity for NMDA receptors. Memantine binds to a cationic cochannel on the NMDA receptor that overlaps with the magnesium site, thereby inhibiting glutamate binding to the NMDA receptor.^14^ Therefore, memantine can inhibit the overactivation of the NMDA receptor, antagonize the adverse reaction of increased glutamate levels in the brain, and protect neurons from the effects of glutamine‐mediated excitatory toxicity.^27^

There are five neuropathological features of AD, but memantine only works on two of these features, which represents a limitation of memantine treatment for AD. Therefore, we believe that the addition of other drugs that can act on the neuropathological features of AD can enhance or complement memantine function in the treatment of AD.^28^ Combination therapy seems to be a more reasonable approach for the treatment of AD.^29^

### 3.4 Current status of memantine combination therapy

Currently, the FDA has approved five individual drugs and one drug combination for the treatment of AD, with the only drugs approved for combination therapy being donepezil and memantine. Donepezil is an acetylcholinesterase inhibitor (ChEI). The combination therapy of memantine and ChEI, although not approved, is valued by a wide range of researchers. Both authorities, Clinical Practice Guidelines and Scientific Societies, have found the combination of memantine and ChEI to be significantly effective in patients with advanced AD. The reason for this is that the two drugs can compensate for each other's shortcomings in symptomatic treatment and are more effective than monotherapy with five drugs.^24^ The comparison of memantine and cholinesterase combination therapy with cholinesterase monotherapy in restoring cognitive function capacity has yielded different or even opposite results from those of different researchers. The effect of memantine in combination with cholinesterase for AD cannot be determined.^30^ The combination of memantine and donepezil for AD has advantages and disadvantages compared to monotherapy. The advantage is better improvement in cognitive function and higher overall patient assessment scores. The disadvantage is that the patient's body is less adaptable to this combination therapy.^29^ Other studies have shown that patients receiving therapy with ChEI and memantine reported significantly higher rates of side effects compared with memantine monotherapy. Combination therapy still has a long way to go.^31^ A combination of strategies, including P‐DMTS, acousto‐optic stimulation of brain waves, symptomatic treatment, and nerve regeneration, may be useful in the future.^32^

### 3.5 Comparison of memantine with other drugs for AD

#### 3.5.1 Memantine and ChEIs

ChEIs currently approved for the treatment of AD include donepezil, rivastigmine, and galantamine, which are primarily used for the treatment of mild to moderate AD. In contrast, memantine is primarily used for the treatment of moderate to severe AD.^33^ In comparison with ChEIs, memantine is used to treat more advanced AD. In patients with earlier AD, ChEIs are more capable than memantine of alleviating progressive cognitive decline in the body. Cholinergic synapses from the basal forebrain projecting to the cortex are lost, and cholinergic energy is reduced in patients with AD. The loss of cholinergic nerves in the basal forebrain of AD patients results in the denervation of presynaptic cholinergic neurons. ChEIs compensate for the decrease in cholinergic energy in patients by inhibiting acetylcholine catabolism and enhancing interneural message transmission.^34^ Memantine inhibits the excessive activation of NMDA receptors, thereby antagonizing glutamine‐mediated excitotoxicity to neuronal damage. Memantine also enhances cholinergic signaling and, like ChEIs, can reduce acetylcholine deficiency in AD patients. A study that combined data from 80 trials found that the mini‐mental state examination (MMSE) score results for ChEls over 6 months were better than the results for memantine.^35^ It has also been shown that ChEIs and memantine are not effective in restoring cognitive function and relieving neuropsychiatric symptoms in patients with poor overall outcomes.^36^ However, ChEIs and memantine reduce the risk of death in patients with AD, and donepezil has a greater ability to reduce the risk of death than memantine.^34^ High doses of Rivastigmine patches have been shown to be very effective in functional recovery and initial diagnosis. In contrast, the oral form of rivastigmine causes more serious and highly prevalent side effects. Memantine has better acceptability than the oral form of rivastigmine.^19^ ChEI therapy for AD has limited efficacy and can only delay the progression of the disease, but not stop its deterioration. The reason for this is not only the complex neuropathological changes in AD but also the peripheral cholinergic stimulation that limits the dose of cholinesterase administration. It is the organism tolerance problems (such as dizziness, fatigue, muscle cramps, nausea, etc.) that lead to dose restrictiveness. Moreover, with the progression of AD, the presynaptic neurons that release Ach into the synaptic gap progressively dissolve, Ach decreases, and the efficacy of ChEIs diminishes.^24^

#### 3.5.2 Memantine and aducanumab

Extracellular deposition of amyloid‐β is one of the neuropathological features of AD. Amyloid‐beta (Aβ) is sticky, and when it reaches a certain amount in the brain, it aggregates into small protein clumps and then gradually forms plaques. The specific forms of Aβ present in plaques include monomers, oligomers, protofibrils, and insoluble fibers. Of these, oligomers and protofibrils are the most neurotoxic. Aducanumab is a monoclonal IgG1 antibody that binds to Aβ plaques.^37^ Aducanumab captures aggregated Aβ and provides Aβ to microglia for phagocytosis, thereby clearing Aβ.^28^ Aducanumab has provided a new direction to AD treatment as the first disease‐modifying therapy (DMT) approved for the treatment of AD. In addition to aducanumab, many other DMTs are currently being investigated, some of which are in advanced stages of development.^24^ Both ChEls and memantine are only symptomatic treatments for AD and have limited efficacy.^16^ Aducanumab is the only new drug approved for the treatment of AD in the last 20 years, and its emergence is significant. However, the theory that the removal of Aβ plaques restores cognitive function in AD patients has not been fully confirmed by experiments. Many scholars are skeptical of the FDA's approval of aducanumab for reasons including the different results of the two phase 3 trials: EMERGE and ENGAGE. By performing a new analysis of data from studies that have been conclusive, the researchers found that from the results obtained, it is difficult to prove that aducanumab is effective and safe in treating AD and needs to be further explored. In December 2021, the European Medicines Agency denied the approval of aducanumab for AD treatment, resulting in more skepticism as to whether the drug is effective and safe.^38^ Also, aducanumab is expensive: about $28,000 per person per year, making it unaffordable for the average family.^39^ In comparison with aducanumab, memantine is less controversial and less costly but has limited effects.

## 4 MEMANTINE'S THERAPEUTIC POTENTIAL FOR OTHER DISEASES

### 4.1 Ischemic brain injury after cardiac arrest

Memantine, as the only noncompetitive receptor antagonist of NMDA in clinical practice, has received extensive attention from researchers. Further research into the medicinal properties of memantine has revealed additional biological effects that could be considered in clinical studies. Progression from ischemia–reperfusion injury to neuronal cell death after cardiac arrest is a complex process involving a number of key steps, including excitatory toxicity, mitochondrial dysfunction, oxidative stress, intracellular calcium overload, and overactivation of inflammatory response. Excitotoxicity appears early in the ischemia caused by cardiac arrest and the damage to nerve cells is severe and can further aggravate brain damage, so we need to treat it promptly.^40^ Excitotoxicity is associated with excessive activation of excitatory amino acids, mainly glutamate and aspartate. However, memantine can bind to the NMDA receptor, which can inhibit the overactivation of the NMDA receptor and protect neurons from the effects of glutamine‐mediated excitatory toxicity.^41^ Therefore, memantine has certain clinical significance in the neuroprotection of ischemic brain injury after cardiac arrest.

### 4.2 ALI

Macrophages are an important line of defense for the lung against pathogenic microbial invasion and lung injury. Memantine alleviates the lung damage caused by ALI by inhibiting macrophage death.^42^ The formation and progression of ALI are associated with two factors. The first is a direct lung injury that can lead to ALI. The second is a systemic inflammatory response caused by factors such as sepsis, infection, trauma, shock, and major surgery. Systemic inflammation influences the process of ALI formation and progression through neurohumoral factors.^43^ Alveolar macrophages are the main and key white blood cells in the alveolar lumen and are active in phagocytosis, immunity, and secretion. Various inflammatory mediators synthesized and released by alveolar macrophages influence the progression of ALI.^42^ Patients with ALI have overactive alveolar macrophages, leading to cell death and uncontrolled inflammation in the lungs. Further development of inflammation can be inhibited by reducing cellular damage. We can treat ALI by controlling the death signal of alveolar macrophages through drugs, thus inhibiting cell death.^42^, ^44^ Memantine inhibits activation of alveolar macrophage death signaling through inhibition of Ca^2+^ inward flow and subsequent apoptosis‐associated speck‐like protein containing CARD(ASC) oligomerization, thereby inhibiting cell death and reducing inflammation, which has implications for clinical studies of ALI.^42^

### 4.3 Dementia

Dementia refers to a group of brain disorders that affect memory, reasoning, judgment, executive function, practice, visuospatial ability, and language that is not part of delirium or other major mental disorders. Vascular dementia accounts for 15%–20% of all cases of dementia patients and is the second most common type of dementia, just below AD.^45^ The development of vascular cognitive impairment and vascular dementia is associated with the presence of damaging factors such as blockage and narrowing of blood vessels.^46^ Vascular dementia causes multiple higher cortical functions to be impaired. Vascular dementia is associated with cognitive dysfunction. Vascular dementia is caused by reduced blood supply to the brain due to vascular injury. Therefore, patients with cerebrovascular and cardiovascular disease are more likely to develop vascular dementia than normal individuals.^47^ Unlike AD, no drug is clearly effective for vascular dementia, for which there is currently no approved treatment.^45^, ^48^ There is an urgent need to explore the pathogenesis and treatment methods of vascular dementia. However, all four drugs, other than aducanumab approved by the FDA for the treatment of AD, improve cognitive function in patients with vascular dementia.^49^ All four drugs are safe for patients with vascular dementia, but their effects are very limited. Memantine is globally recognized for its ability to enhance cognition in patients with vascular dementia.^50^

Dementia is one of the most serious symptoms of Parkinson's disease, as it increases the patient's burden of life and reduces mobility and vitality. Early detection and diagnosis of Parkinson's disease dementia are an important part of clinical care and currently need to be vigorously developed. Parkinson's disease and dementia have been found to be associated in a Sydney multicenter. People with Parkinson's disease are much more likely to develop dementia than those who do not have Parkinson's disease; for example, patients with Parkinson's disease have a 75% probability of developing dementia within 10 years of diagnosis and up to 83% incidence within 20 years.^51^ However, there may be an underdiagnosis of Parkinson's disease dementia in neurological clinics, which often leads to further exacerbation of the disease. Parkinson's disease dementia is a systemic disorder that affects memory and cognition through a network of connections between the cortex and cortex‐related areas.^51^ Damaged neurons in patients with Parkinson's disease and dementia are sensitive to glutamate excitatory toxicity, but excess glutamate is not observed. Memantine can inhibit excitatory toxicity, so it has certain significance in the treatment of Parkinson's disease and dementia (Table 1).

**Table 1:** Summary of the application of memantine in some diseases, except for Alzheimer's disease (AD).

Disease	Etiological factor	The role of memantine
Acute lung injury	Direct lung injury, indirect systemic inflammation	Memantine inhibits macrophage Nlrp3 inflammasome activation and focal degeneration by inhibiting Ca^2+^ influx and subsequent apoptosis‐associated speck‐like protein containing CARD oligomerization
Vascular dementia	Small‐vessel disease, reduced blood flow to brain tissue,	Enhance cognitive function
Parkinson's disease and dementia	A systemic disorder, complex	Inhibit excitatory toxicity

Memantine is also being studied in clinical trials for ischemic stroke, CNS trauma, epilepsy, glaucoma, amyotrophic lateral sclerosis (ALS), and other diseases. In sum, the application of memantine is promising.

## 5 CONCLUSIONS AND PROSPECTS

This article reviews the history of memantine, the mechanism, and limitations of memantine for AD, and memantine and combination therapy, and compares memantine with other drugs approved by the FDA for the treatment of AD. AD places a tremendous burden on families, society, and the world. According to statistics, the cost of patient care due to AD and related dementia diseases was approximately $290 billion in 2019.^52^ AD is a complex neurodegenerative disease with five major neuropathological features, but the specific mechanisms of formation of these features have not been fully clarified.^53^ We need to continue to explore the molecular mechanisms that cause AD and then explore new therapeutic approaches based on the mechanisms. Memantine was initially studied as an antiglycemic drug and was overlooked because it had little effect. It was not until after 1892 that the value of memantine was considered. It is very difficult to produce an entirely new drug, and we can explore more of the value of currently available drugs in other diseases. For example, memantine has been used to treat many other diseases (ischemic brain injury after cardiac arrest, ALI, dementia, glaucoma, etc.). Also, exploring the mechanism of memantine in each disease will help us to explore other clinical effects of memantine based on the mechanism. Memantine has been approved by the FDA for the treatment of moderate to severe AD, but has limited efficacy and many side effects. Combination therapy is one of the possible ways to improve the outcome of memantine treatment, but only donepezil and memantine are currently approved by the FDA. We can explore more strategic combinations of P‐DMTS, symptomatic treatment, and nerve regeneration. We compared ChEIs and aducanumab with memantine. Memantine is more suitable than ChEls for the treatment of advanced AD. Aducanumab provides a new avenue in AD treatment compared to memantine, but its efficacy and safety are currently more controversial in the scientific community, and it is expensive and unaffordable for long‐term use for the average family. After comparison, memantine still has irreplaceable significance in AD treatment. We can learn from the development of memantine that to make the best use of a drug, one has to understand its molecular mechanism. At the same time, we should continue to study the pathological mechanisms of AD and develop new treatment methods according to pathological mechanisms. Memantine, as a classical drug for the treatment of AD, has great potential in clinical research. It is believed that with the efforts of all people, the pathological mechanisms of AD will be elucidated and AD will become a curable disease in the future. We can also explore completely new uses for memantine in the future.

## AUTHOR CONTRIBUTIONS

Bin‐Can Tang wrote the article. Ya‐Ting Wang and Jie Ren revised the article.

## CONFLICT OF INTEREST STATEMENT

The authors declare no conflict of interest.

## ETHICS STATEMENT

Not applicable.

## Data Availability

Data sharing is not applicable to this article as no data sets were generated or analyzed during the current study.
